# Association Between Average Plasma Potassium Levels and 30-day Mortality During Hospitalization in Patients with COVID-19 in Wuhan, China

**DOI:** 10.7150/ijms.50965

**Published:** 2021-01-01

**Authors:** Shengcong Liu, Long Zhang, Haoyu Weng, Fan Yang, Han Jin, Fangfang Fan, Xizi Zheng, Hongyu Yang, Haichao Li, Yan Zhang, Jianping Li

**Affiliations:** 1 Department of Cardiology, Peking University First Hospital, Beijing, China; 2 Institute of Cardiovascular Disease, Peking University First Hospital, Beijing, China; 3 Department of Nephrology, Peking University First Hospital, Beijing, China; 4 Department of Respiratory and Critical Care Medicine, Peking University First Hospital, Beijing, China; 5 Key Laboratory of Molecular Cardiology Sciences of the Ministry of Education, Peking University Health Science Center, Beijing, China

**Keywords:** COVID-19, Potassium, Mortality, Prognosis

## Abstract

**Background:** Coronavirus disease 2019 (COVID-19) has resulted in more than 610,000 deaths worldwide since December 2019. Given the rapid deterioration of patients' condition before death, markers with efficient prognostic values are urgently required. During the treatment process, notable changes in plasma potassium levels have been observed among severely ill patients. We aimed to evaluate the association between average plasma potassium (K_a_^+^) levels during hospitalization and 30-day mortality in patients with COVID-19.

**Methods:** Consecutive patients with COVID-19 hospitalized in the Zhongfaxincheng branch of Tongji Hospital in Wuhan, China from February 8 to 28, 2020 were enrolled in this study. We followed patients up to 30 days after admission.

**Results:** A total of 136 patients were included in the study. The average age was 62.1±14.6 years and 51.5% of patients were male. The median baseline potassium level was 4.3 (3.9-4.6) mmol/L and K_a_^+^ level during hospitalization was 4.4 (4.2-4.7) mmol/L; the median number of times that we measured potassium was 4 (3-5). The 30-day mortality was 19.1%. A J-shaped association was observed between K_a_^+^ and 30-day mortality. Multivariate Cox regression showed that compared with the reference group (K_a_^+^ 4.0 to <4.5 mmol/L), 30-day mortality was 1.99 (95% confidence interval [CI]=0.54-7.35, P=0.300), 1.14 (95% CI=0.39-3.32, P=0.810), and 4.14 (95% CI=1.29-13.29, P=0.017) times higher in patients with COVID-19 who had K_a_^+^ <4.0, 4.5 to <5.0, and ≥5.0 mmol/L, respectively.

**Conclusion:** Patients with COVID-19 who had a K_a_^+^ level ≥5.0 mmol/L had a significantly increased 30-day mortality compared with those who had a K_a_^+^ level 4.0 to <4.5 mmol/L. Plasma potassium levels should be monitored routinely and maintained within appropriate ranges in patients with COVID-19.

## Introduction

Coronavirus disease 2019 (COVID-19), caused by severe acute respiratory syndrome coronavirus 2 (SARS-COV-2) infection, has spread to more than 200 countries worldwide since it was first reported in December 2019. According to a World Health Organization report on July 21, 2020, over 15 million confirmed cases of COVID-19 have been reported in many countries, including more than 610,000 deaths. This situation represents the largest public health crisis in the world. Compared with previous coronavirus-related diseases like severe acute respiratory syndrome and Middle East respiratory syndrome, COVID-19 is characterized by a higher infection rate and lower mortality rate. However, given the large denominator, the absolute number of deaths is still staggeringly high. It has been estimated that the unadjusted global case fatality rate of COVID-19 is 4%-5%^1^. However, the case fatality rate varies notably from country to country, for undefined reasons.

A huge challenge in reducing the case fatality rate is the unpredictable nature of the disease. Patients may present with mild symptoms in the early stages and then experience dramatic exacerbation in later stages or during the process of recovery. Therefore, identifying individual characteristics that can predict poor outcomes has been a priority for investigators. Several clinical characteristics, including older age, concomitant cardiovascular or respiratory diseases, diabetes, and cancer, have been proposed as being related to poor clinical outcomes. Laboratory abnormalities, including markers of inflammation, coagulation, and cardiac injury have also been correlated with disease severity^2^, ^3^. However, at present, factors related to the prognosis of COVID-19 remain unclear. Previous studies have shown that significant changes in potassium are often associated with poor outcomes in many diseases^4^-^6^. Recent studies suggest an increased incidence of electrolyte imbalance in patients with severe COVID-19^7^. However, the relationship between plasma potassium and the prognosis of patients with COVID-19 has not been reported. Therefore, in the present study, we retrospectively analyzed the association of average plasma potassium (K_a_^+^) levels and 30-day mortality in hospitalized patients with COVID-19.

## Methods

### Study participants

In early February, a medical team from Peking University First Hospital, People's Hospital, and Third Hospital was sent to Wuhan, China to support treatment of local patients with COVID-19. Team members were assigned to treat patients in the Zhongfaxincheng branch of Tongji Hospital in Wuhan. From February 8 to 28, a total of 138 patients were transferred to our wards. All patients were confirmed to be infected with SARS-CoV-2 in secondary-level hospitals or Fangcang hospitals and were transported to our ward owing to aggravation of the disease and critical care demands. Among them, two patients had no records of outcomes. Thus, we enrolled 136 participants in this study.

Patients were diagnosed according to the World Health Organization interim guidance and were clinically classified based on the "Diagnosis and Treatment Protocol for Novel Coronavirus Pneumonia (Trial Version 7)" issued by the National Health Commission of the People's Republic of China. Severe cases were defined as having one of the following: (i) respiratory rate >30 breaths/min, (ii) oxygen saturation ≤93%, or (iii) partial pressure of oxygen/fraction of inspired oxygen ratio ≤300 mmHg. Critical cases were defined as having at least one of the following: shock, respiratory failure requiring mechanical ventilation, or extrapulmonary organ failure requiring intensive care. The Ethics Committees of Peking University First Hospital approved the study.

### Patient clinical data collection

General clinical data on the enrolled patients were collected from the electronic medical record system and included demographics (age and sex), clinical data (signs, symptoms, chronic medical illnesses, treatment, and clinical outcomes), and laboratory findings. The clinical outcomes were defined as death within 30 days after hospital admission. The last day of follow-up was 20 March 2020.

All plasma potassium (K^+^) values measured during hospitalization for each patient were recorded and used to calculate the K_a_^+^ level for each patient. To avoid errors in plasma K^+^ measurement, plasma samples were drawn from a central or arterial line containing no K^+^ solution. The specimens were collected in a heparin-coated plastic tube and transferred directly to the central laboratory. The plasma K^+^ level was then measured using an ion-selective electrode.

All patients were treated as per the "Diagnosis and Treatment Protocol for Novel Coronavirus Pneumonia (Trial Version 7)". All patients with abnormal plasma potassium levels were given standard potassium supplementation or potassium reduction therapy. The clinical outcomes (all-cause mortality) were monitored.

### Statistical analysis

Data are presented as mean ± standard deviation (SD) for continuous variables that were normally distributed and as median and interquartile range (IQR) for continuous variables that were not normally distributed. Categorical variables are expressed as number and percentage. Proportions were compared using the χ^2^ test. Mean values were compared using *t*-tests when the data were normally distributed and the Mann-Whitney U test otherwise. Multivariate Cox regression, including sex, age, history of chronic kidney disease, pulse oxygen saturation (SpO_2_), lymphocyte count, creatinine, and D-dimer levels, was used to determine the association of the average plasma K^+^ level during hospitalization with clinical outcomes. Hazard ratios (HRs) and 95% confidence intervals (CIs) were determined to indicate the effect. Statistical significance was set to P value <0.05. All statistical analyses were performed using EmpowerStats 2.20 (http://www.empowerstats.com/en/index.html) and R 3.4.3 (https://www.r-project.org/).

## Results

### Baseline demographic and clinical characteristics of patients with COVID-19

Baseline clinical characteristics of the enrolled patients are shown in Table 1. The average patient age was 62.1±14.6 years old, 70 (51.5%) patients were male, and 30 (22.1%) had a smoking history. The average body mass index (BMI) was 24.2±3.0 kg/m^2^. In total, 62 (45.6%) patients had hypertension, which was the most common comorbidity. Coronary artery disease, chronic pulmonary disease, diabetes, and chronic kidney disease were present in 26 (19.1%), 21 (15.4%), 27 (19.9%), and 9 (6.6%) patients, respectively.

Fever (117 [86.0%]) was the most common symptom, followed by cough (114 [83.8%]), shortness of breath (90 [66.2%]), sputum production (84 [61.8%]), fatigue (84 [61.8%]), and muscle ache (64 [47.1%]). Digestive system symptoms were also common, such as nausea (46 [33.8%]), diarrhea (46 [33.8%]), vomiting (28 [20.6%]), and stomachache (25 [18.4%]) (Table 1).

We observed an increase in the average respiratory rate (24.9±9.4 bpm) and heart rate (97.3±18.1 bpm). The average body temperature was 36.7±0.7 °C and median SpO_2_ was 95.0% (89.0%-97.0%) at admission. In total, 96 (70.6%) patients received antiviral treatment before admission. 94 (69.1%), 16 (11.8%), and 12 (9.0%) patients received antibiotic treatment, glucocorticoids, and intravenous immunoglobulin therapy before admission, respectively.

Laboratory tests showed that the median level of baseline K^+^ was 4.3 (3.9-4.6) mmol/L and K_a_^+^ was 4.4 (4.2-4.7) mmol/L; the median number of times that we measured K^+^ was 4 (3-5). The median level of white blood cells (WBC), lymphocytes, and platelets was 5.5 (4.4-7.7)×10^9^/L, 0.9 (0.6-1.4)×10^9^/L, and 227.5 (159.0-291.2)×10^9^/L, respectively. The median creatinine level was 73.5 (58.0-91.0) µmol/L and estimated glomerular filtration rate (eGFR) level was 77.7 (57.1-94.2) mL/min/1.73m^2^. Elevated median high-sensitivity C-reactive protein (35.7 [6.3-82.0] mg/L), fibrinogen (5.2 [4.0-6.4] g/L), and D-dimer (1.3 [0.5-2.5] µg/mL) levels were found.

Patients were divided into four groups according to K_a_^+^ level: <4.0 mmol/L, 4-4.5 mmol/L, 4.5-5.0 mmol/L, and ≥5.0 mmol/L. There was no significant difference in age, sex, smoking history, BMI, vital signs, and symptoms among the four groups. Chronic medical illnesses and treatments before admission were similar among the four groups, except for a history of chronic kidney disease (P<0.001).

Median levels of lymphocytes (P=0.033) and renal function indicators were significantly different among the four groups. Creatinine (P<0.001) and blood urea nitrogen (P<0.001) were higher in patients with K_a_^+^ ≥5.0 mmol/L, and eGFR (P<0.001) was significantly lower in the ≥5.0 mmol/L group. We observed a trend of decrease in platelet count (P=0.055) and an increase in the level of N-terminal-pro-B-type natriuretic peptide (P=0.061) in patients with K_a_^+^ ≥5.0 mmol/L compared with patients who had lower K_a_^+^ levels, but there was no significant difference. Other laboratory tests, including sodium, chlorine, bicarbonate, WBC, hemoglobin, neutrophils, liver function indicators, cardiac troponin I, coagulation function indicators, and high-sensitivity C-reactive protein, were similar among the four groups (Table 1).

### Associations of plasma potassium level with 30-day mortality during hospitalization

**Figure 1:**
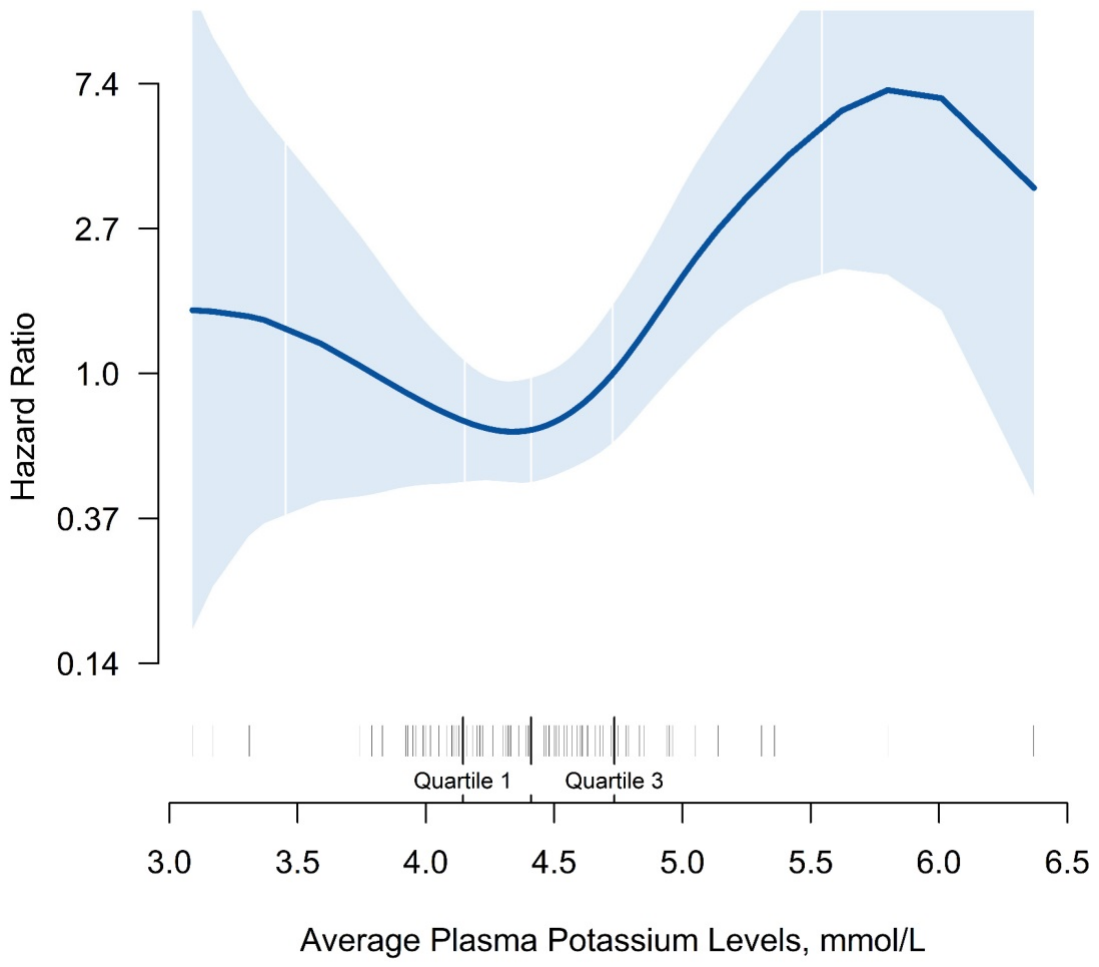
Smooth curve of average plasma potassium level with 30-day mortality during hospitalization in patients with COVID-19

**Figure 2:**
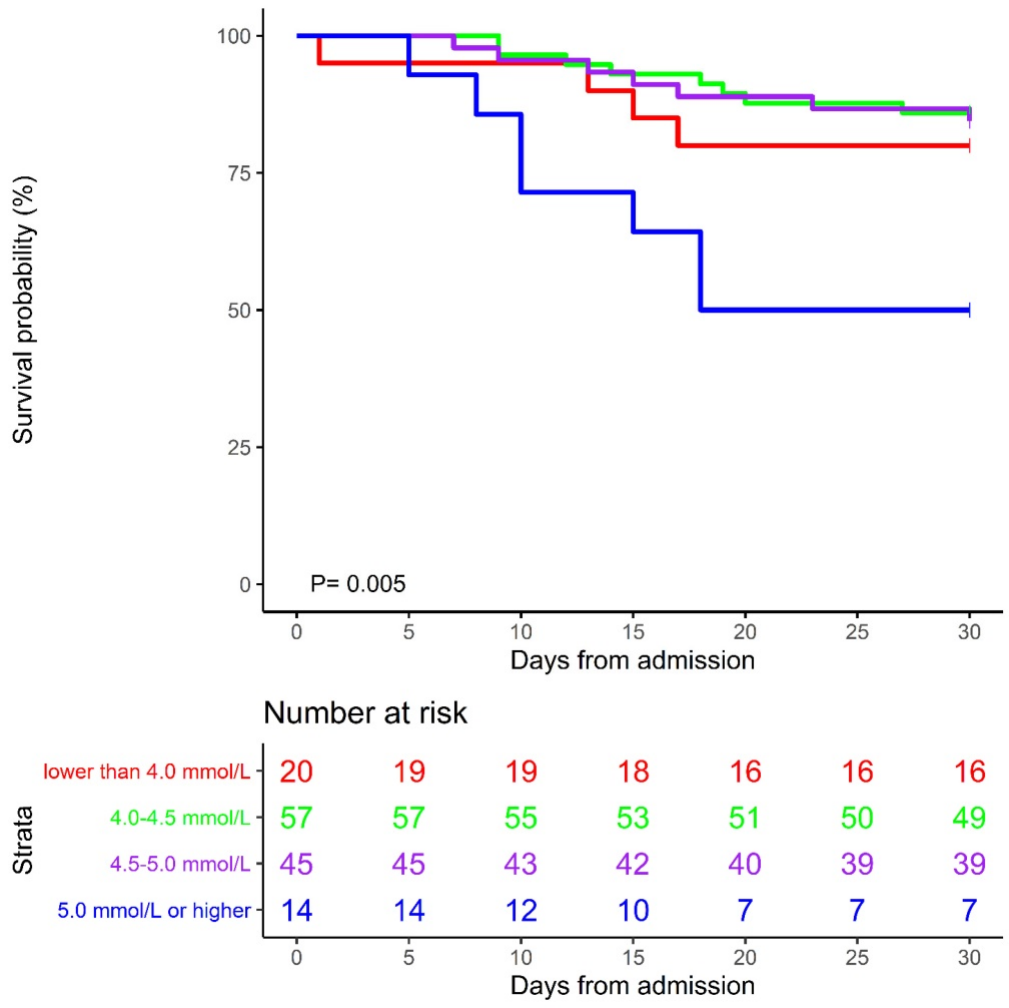
Kaplan-Meier curves of 30-day mortality

In this study, the overall 30-day mortality was 19.1% (26/136). A J-shaped association of K_a_^+^ levels with 30-day mortality was observed. Patients with lower or higher K_a_^+^ levels had an increasing trend of 30-day mortality, with the minimum level 4.0 to <4.5 mmol/L (Figure 1). Kaplan-Meier curves showed that mortality was increased among patients with higher potassium levels. Patients with K_a_^+^ ≥5.0 mmol/L had the highest HRs in 30-day mortality, compared with the remaining patients (P=0.005, Figure 2). In the multivariate Cox regression model adjusted for potential confounders, 30-day mortality was 3.14 (95% CI=1.29-13.29, P=0.017) times higher in patients with K_a_^+^ level ≥5.0 mmol/L than in the reference group (with K_a_^+^ 4.0 to <4.5 mmol/L), with statistical significance. There was also a higher trend for risk of mortality in both patients with K_a_^+^ levels <4.0 mmol/L (HR=1.99, 95% CI=0.54-7.35, P=0.300) and 4.5 to <5.0 mmol/L (HR=1.14, 95% CI=0.39-3.32, P=0.810) (Table 2).

## Discussion

The main finding of this study was that an abnormal plasma K^+^ level during hospitalization was associated with adverse patient outcomes. The risk of 30-day mortality during hospitalization was significantly increased in patients with COVID-19 who had K_a_^+^ ≥5.0 mmol/L, as compared with the 4.0 to <4.5 mmol/L group. These findings support the evidence that plasma K^+^ has a significant impact on outcomes in patients with COVID-19.

At present, the COVID-19 pandemic is causing a global public health crisis. The incidence of acute respiratory distress syndrome and critical illness requiring admission to the intensive care unit (ICU) is 17%-29% and 23%-32%, respectively, according to reports from hospitals in Wuhan^8^-^10^. The mortality rate among critically ill patients ranges from 38% to 62%^11^-^13^. In our study, the patient mortality rate was 19.1% (26/136). Considering that some patients with COVID-19 have rapid progression of disease, early identification of patients with a higher risk of critical illness is crucial. Studies have shown that patients with older age, more comorbidities, elevated D-dimer levels, and decreased lymphocytes have poorer clinical outcomes^8^, ^10^, ^14^, ^15^. However, the factors affecting prognosis still need to be explored.

In many previous studies, a J-shaped or U-shaped correlation was found between potassium level and prognosis of the disease. Hypokalemia or hyperkalemia are both poor prognostic factors. A prospective cohort study showed that critically ill patients in the medical ICU with abnormal K^+^ levels had a higher incidence of ICU mortality than patients with normal K^+^ levels^4^. A prospective observational study showed that increased serum potassium was an independent predictor of mortality in patients with severe community-acquired pneumonia^16^. A systematic review and meta-analysis indicated that both lower (<3.5 mEq/L) and higher (4.5 mEq/L) serum potassium levels are associated with an increased risk of mortality of patients with acute myocardial infarction^5^. A large retrospective analysis of patients in the ICU indicated the lowest mortality was observed in patients with mean potassium concentrations between 3.5 and <4.0 mmol/L^17^. To the best of our knowledge, the association between plasma potassium and mortality in patients with COVID-19 has rarely been investigated. A recent study showed that COVID-19 severity is associated with lower concentrations of potassium^7^. In the present study, we found that the risk of death was significantly increased in patients with COVID-19 who had K_a_^+^ ≥5.0 mmol/L, as compared with the 4.0 to <4.5 mmol/L group. Similarly, a J-shaped association was observed between K_a_^+^ level and the mortality rate, with the minimum level 4.0 to <4.5 mmol/L. However, we did not find a significant risk of hypokalemia, which may be related to the sample size.

The exact pathophysiological mechanisms by which hyperkalemia is related to higher mortality in patients with COVID-19 are uncertain. However, several possible mechanisms may partly explain recent findings. First, potassium levels modify the electrophysiological properties of the resting membrane potential in the myocardium and subsequently contribute to the occurrence of ventricular arrhythmia^18^. Hyperkalemia is known to decrease ventricular excitability and precipitate complete heart block and sinus arrest. Elevated serum potassium levels are also associated with an increased risk of significant bradyarrhythmia^19^. Second, severely and critically ill patients, who are frequently affected by organ dysfunction and often receive multiple medications that affect K^+^ regulation, are at high risk of developing abnormal K^+^ levels^20^. Third, abnormal plasma potassium may be one of the manifestations of acid-base balance disorder, which suggests severe acute respiratory distress syndrome^21^.

The advantage of this study is that we used average patient plasma potassium levels during hospitalization, which reflects the overall information of plasma potassium and also reflects the effect of potassium regulation treatment.

There are also some limitations in this study. First, this was a single-center retrospective study that included a small number of patients. In the future, the sample size should be expanded to include other populations, especially focusing on the impacts of hypokalemia. Second, the intervals of plasma potassium measurement were not uniform, which cannot reflect the influence of change trends; however, the average level of multiple tests reflects the overall information of plasma potassium, which has also been proved to be a valuable indicator^4^. Third, there is a lack of plasma potassium treatment information. However, in this study, we focused on the average level of plasma potassium, which also reflects the effect of potassium modulation treatment. The results of this study need to be confirmed in additional populations and multicenter randomized controlled trials in the future.

## Conclusion

Patients with COVID-19 who had an average plasma potassium level ≥5.0 mmol/L had a significantly increased 30-day mortality compared with those who had an average plasma potassium level 4.0 to <4.5 mmol/L. Plasma potassium levels should be monitored routinely and maintained within appropriate ranges in patients with COVID-19.

## Figures and Tables

**Table 1:** Baseline clinical characteristics of 136 patients with COVID-19

Characteristics	Total (n=136)	K_a_^+^<4 (n=20)	K_a_^+^>=4, <4.5 (n=57)	K_a_^+^>=4.5, <5 (n=45)	K_a_^+^>=5 (n=14)	P-value
Age, mean±SD, y	62.1 ± 14.6	61.0 ± 14.0	62.6 ± 15.6	60.4 ± 14.5	67.2 ± 10.7	0.478
Male, %	70 (51.5%)	6 (30.0%)	28 (49.1%)	26 (57.8%)	10 (71.4%)	0.080
Smoking, %	30 (22.1%)	4 (20.0%)	11 (19.3%)	11 (24.4%)	4 (28.6%)	0.850
BMI, mean±SD , kg/m^2^	24.2±3.0	23.3 ± 2.5	24.8 ± 3.6	23.9 ± 2.4	24.2 ± 2.3	0.247
Chronic medical illness						
Hypertension	62 (45.6%)	8 (40.0%)	30 (52.6%)	19 (42.2%)	5 (35.7%)	0.542
Diabetes Mellitus	27 (19.9%)	4 (20.0%)	13 (22.8%)	8 (17.8%)	2 (14.3%)	0.871
Coronary Heart Disease	26 (19.1%)	3 (15.0%)	13 (22.8%)	8 (17.8%)	2 (14.3%)	0.805
Chronic Pulmonary Disease	21 (15.4%)	2 (10.0%)	8 (14.0%)	9 (20.0%)	2 (14.3%)	0.736
Chronic Kidney Disease	9 (6.6%)	0 (0.0%)	3 (5.3%)	0 (0.0%)	6 (42.9%)	<0.001
Signs and symptoms						
SBP at admission, mean±SD, mmHg	133.2 ± 22.4	128.3 ± 20.1	135.3 ± 23.4	129.2 ± 21.5	145.6 ± 20.9	0.074
DBP at admission, mean±SD, mmHg	82.5 ± 14.7	82.5 ± 12.9	81.1 ± 14.3	82.6 ± 16.1	88.6 ± 14.3	0.438
Heart rate at admission, mean±SD, bpm	97.3 ± 18.1	94.8 ± 19.4	100.3 ± 18.7	94.2 ± 15.8	98.5 ± 20.4	0.344
Temperature at admission, mean±SD, °C	36.7 ± 0.7	36.6 ± 0.7	36.8 ± 0.9	36.6 ± 0.5	36.8 ± 0.8	0.568
Respiratory rate at admission, mean±SD, bpm	24.9 ± 9.4	23.5 ± 6.1	24.4 ± 10.4	25.7 ± 10.2	26.2 ± 6.9	0.840
SpO_2_ at admission, median (IQR), %	95.0 (89.0-97.0)	94.5 (90.8-96.2)	95.0 (90.0-98.0)	95.0 (88.0-97.0)	96.0 (85.0-97.0)	0.736
Fever, %	117 (86.0%)	18 (90.0%)	48 (84.2%)	39 (86.7%)	12 (85.7%)	0.933
Cough, %	114 (83.8%)	16 (80.0%)	47 (82.5%)	39 (86.7%)	12 (85.7%)	0.897
Sputum production, %	84 (61.8%)	11 (55.0%)	37 (64.9%)	26 (57.8%)	10 (71.4%)	0.686
Shortness of breath, %	90 (66.2%)	12 (60.0%)	40 (70.2%)	28 (62.2%)	10 (71.4%)	0.745
Chest pain, %	25 (18.4%)	4 (20.0%)	9 (15.8%)	10 (22.2%)	2 (14.3%)	0.828
Sore throat, %	29 (21.3%)	3 (15.0%)	11 (19.3%)	14 (31.1%)	1 (7.1%)	0.182
Diarrhea, %	46 (33.8%)	8 (40.0%)	33 (57.9%)	22 (48.9%)	7 (50.0%)	0.546
Nausea, %	46 (33.8%)	8 (40.0%)	19 (33.3%)	16 (35.6%)	3 (21.4%)	0.713
Vomiting, %	28 (20.6%)	5 (25.0%)	14 (24.6%)	8 (17.8%)	1 (7.1%)	0.466
Stomachache, %	25 (18.4%)	5 (25.0%)	10 (17.5%)	8 (17.8%)	2 (14.3%)	0.855
Muscle ache, %	64 (47.1%)	9 (45.0%)	27 (47.4%)	24 (53.3%)	4 (28.6%)	0.446
Fatigue, %	84 (61.8%)	14 (70.0%)	36 (63.2%)	27 (60.0%)	7 (50.0%)	0.682
Treatment before admission						
Antiviral treatment, %	96 (70.6%)	12 (60.0%)	39 (68.4%)	36 (80.0%)	9 (64.3%)	0.334
Antibiotic treatment, %	94 (69.1%)	14 (70.0%)	38 (66.7%)	33 (73.3%)	9 (64.3%)	0.874
Intravenous immunoglobulin therapy, %	12 (9.0%)	1 (5.3%)	8 (14.0%)	1 (2.3%)	2 (14.3%)	0.180
Glucocorticoids, %	16 (11.8%)	2 (10.0%)	8 (14.0%)	4 (8.9%)	2 (14.3%)	0.853
Laboratory test on admission, median (IQR)						
Potassium	4.3 (3.9-4.6)	3.6 (3.4-3.9)	4.2 (3.9-4.4)	4.5 (4.2-4.8)	4.9 (4.6-5.3)	<0.001
Sodium	140.2 (137.4-142.5)	140.9 (139.2-142.6)	139.2 (137.2-141.8)	140.9 (137.5-143.5)	139.8 (135.9-143.5)	0.609
Chloride	101.0 (98.1-103.2)	99.9 (94.5-102.3)	100.0 (97.5-103.2)	101.4 (99.9-103.6)	101.1 (98.4-102.7)	0.713
Bicarbonate	25.1 (22.8-26.7)	25.2 (22.9-27.1)	25.0 (23.0-26.2)	25.1 (23.0-27.1)	23.1 (21.1-26.4)	0.441
WBC, × 10⁹ per L	5.5 (4.4-7.7)	5.5 (3.7-7.4)	5.8 (4.5-7.0)	5.3 (4.4-8.3)	5.3 (4.6-7.4)	0.954
Hemoglobin, g/L	124.0 (115.0-138.0)	124.0 (115.8-130.0)	122.0 (115.0-130.0)	130.0 (117.0-140.0)	125.0 (100.2-141.5)	0.163
Platelets, × 10⁹ per L	227.5 (159.0-291.2)	173.5 (153.8-236.0)	230.0 (155.0-280.0)	250.0 (201.0-312.0)	175.5 (151.8-241.5)	0.055
Neutrophils, × 10⁹ per L	4.0 (2.7-6.0)	4.0 (2.8-5.8)	4.3 (3.0-5.8)	3.5 (2.5-6.6)	4.0 (2.8-6.4)	0.831
Lymphocytes, × 10⁹ per L	0.9 (0.6-1.4)	0.8 (0.6-1.0)	0.9 (0.6-1.4)	1.3 (0.8-1.6)	0.8 (0.4-1.1)	0.033
ALT, U/L	22.0 (16.0-40.0)	21.0 (13.5-38.2)	22.0 (15.0-40.0)	23.0 (18.0-37.0)	19.5 (12.5-43.8)	0.842
AST, U/L	27.5 (18.0-40.2)	22.5 (18.0-39.8)	28.0 (19.0-39.0)	26.0 (18.0-38.0)	39.5 (20.5-54.5)	0.305
TBIL, µmol/L	9.5 (7.0-12.8)	10.2 (7.0-14.2)	9.5 (6.8-13.4)	9.5 (7.3-10.9)	8.9 (6.9-13.0)	0.806
DBIL, µmol/L	4.2 (3.1-5.8)	4.5 (3.2-7.0)	4.6 (3.1-6.3)	3.9 (3.1-5.1)	4.0 (3.0-6.0)	0.502
Albumin, g/L	34.3 (30.8-37.7)	34.5 (31.3-38.2)	34.0 (30.5-37.7)	33.7 (30.7-37.0)	34.9 (33.5-37.7)	0.878
Creatinine, µmol/L	73.5 (58.0-91.0)	57.5 (52.0-74.2)	75.0 (60.0-94.0)	72.0 (58.0-87.0)	103.0 (78.0-144.8)	<0.001
Blood urea nitrogen, mmol/L	4.6 (3.3-6.5)	3.8 (3.0-5.0)	4.7 (3.1-6.5)	4.3 (3.4-6.0)	9.1 (5.3-11.6)	<0.001
eGFR, ml/min/1.73m^2^	77.7 (57.1-94.2)	93.7 (74.6-100.3)	79.8 (52.7-90.1)	78.0 (58.7-93.8)	51.1 (29.8-70.0)	<0.001
cTnI, pg/mL	4.7 (2.2-10.2)	3.9 (2.4-17.4)	5.3 (2.5-10.2)	3.9 (1.9-7.7)	12.2 (2.7-49.6)	0.342
NT-pro-BNP, pg/mL	180.0 (67.0-456.5)	100.0 (51.5-386.5)	193.0 (75.0-370.0)	134.5 (66.2-374.0)	652.5 (172.2-888.8)	0.061
PT, s	14.1 (13.5-14.7)	14.1 (13.7-14.6)	14.0 (13.4-14.8)	14.0 (13.5-14.4)	14.2 (13.6-15.3)	0.654
APTT, s	40.3 (36.3-44.7)	40.7 (36.7-47.0)	40.2 (36.3-43.3)	39.7 (35.7-44.3)	44.4 (36.0-47.6)	0.611
Fibrinogen, g/L	5.2 (4.0-6.4)	5.2 (4.1-6.2)	5.0 (3.9-6.2)	5.4 (4.0-6.8)	5.2 (4.5-6.3)	0.840
D-dimer, µg/mL	1.3 (0.5-2.5)	1.2 (0.4-2.5)	1.2 (0.5-2.2)	1.2 (0.6-2.5)	1.8 (0.6-7.2)	0.293
Hypersensitive C-reactive protein, mg/dL	35.7 (6.3-82.0)	65.0 (18.2-120.2)	31.0 (7.6-87.5)	25.0 (4.4-46.2)	54.9 (13.8-94.3)	0.455

Abbreviations: SD: standard deviation; K_a_^+^: average plasma potassium; BMI: body mass index; SBP: systolic blood pressure; DBP: diastolic blood pressure; SpO2: pulse oxygen saturation; IQR: interquartile range; WBC: white blood cell; ALT: alanine aminotransferase; AST: aspartate aminotransferase; TBIL: total bilirubin; DBIL: direct bilirubin; eGFR: estimated glomerular filtration rate; cTnI: cardiac troponin I; NT-pro-BNP: N-terminal-pro-B-type natriuretic peptide; PT: prothrombin time; APTT: activated partial thromboplastin time.

**Table 2:** Multivariate Cox regression of average plasma potassium level with 30-day mortality during hospitalization in patients with COVID-19

Variables	30-day death	Non-adjusted		Adjust*	
	n/N (%)	HR (95% CI)	P-value	HR (95% CI)	P-value
K_a_^+^>=4, <4.5	8/57 (14.0%)	1.0	-	1.0	-
K_a_^+^<4	4/20 (20.0%)	1.52 (0.46, 5.04)	0.495	1.99 (0.54, 7.35)	0.300
K_a_^+^>=4.5, <5	7/45 (15.6%)	1.12 (0.41, 3.09)	0.825	1.14 (0.39, 3.32)	0.810
K_a_^+^>=5	7/14 (50.0%)	4.72 (1.71, 13.05)	0.003	4.14 (1.29, 13.29)	0.017

* Adjusted for: sex, age, chronic kidney disease, pulse oxygen saturation, lymphocytes, creatinine, and D-dimer. Abbreviations: HR: hazard ratio; CI: confidence interval; K_a_^+^: average plasma potassium.

## Abbreviations

COVID-19: coronavirus disease 2019
SARS-COV-2: severe acute respiratory syndrome coronavirus 2
K^+^: plasma potassium
K_a_^+^: average plasma potassium
SD: standard deviation
IQR: interquartile range
HR: hazard ratio
CI: confidence interval
BMI: body mass index
SBP: systolic blood pressure
DBP: diastolic blood pressure
SpO_2_: pulse oxygen saturation
WBC: white blood cell
ALT: alanine aminotransferase
AST: aspartate aminotransferase
TBIL: total bilirubin
DBIL: direct bilirubin
eGFR: estimated glomerular filtration rate
cTnI: cardiac troponin I
NT-pro-BNP: N-terminal-pro-B-type natriuretic peptide
PT: prothrombin time
APTT: activated partial thromboplastin time
